# Neuropathic pain after brachial plexus avulsion - central and peripheral mechanisms

**DOI:** 10.1186/s12883-015-0329-x

**Published:** 2015-05-04

**Authors:** Manoel Jacobsen Teixeira, Matheus Gomes da S da Paz, Mauro Tupiniquim Bina, Scheila Nogueira Santos, Irina Raicher, Ricardo Galhardoni, Diego Toledo Fernandes, Lin T Yeng, Abrahão F Baptista, Daniel Ciampi de Andrade

**Affiliations:** Pain Center, Department of Neurology, University of São Paulo, São Paulo, Brazil; Pain Center, Instituto do Câncer de São Paulo, São Paulo, Brazil; Service of Interdisciplinary Neuromodulation (SIN), Department and Institute of Psychiatry, University of São Paulo, São Paulo, Brazil; Functional Electrostimulation Laboratory, Federal University of Bahia, São Paulo, Brazil; Centro de Dor, Instituto de Ortopedia e Traumatologia, University of São Paulo, São Paulo, Brazil; Divisão de Clínica Neurocirúrgica do Hospital das Clínicas da FMUSP, Secretaria da Neurologia, Instituto Central, Av. Dr. Enéas de Carvalho Aguiar, 255, 5° andar, sala 5084 – Cerqueira César, 05403-900 São Paulo, SP Brazil

**Keywords:** Plexus avulsion, Neuropathic pain, Chronic pain, Trauma, Brachial plexus

## Abstract

**Review:**

The pain that commonly occurs after brachial plexus avulsion poses an additional burden on the quality of life of patients already impaired by motor, sensory and autonomic deficits. Evidence-based treatments for the pain associated with brachial plexus avulsion are scarce, thus frequently leaving the condition refractory to treatment with the standard methods used to manage neuropathic pain. Unfortunately, little is known about the pathophysiology of brachial plexus avulsion. Available evidence indicates that besides primary nerve root injury, central lesions related to the abrupt disconnection of nerve roots from the spinal cord may play an important role in the genesis of neuropathic pain in these patients and may explain in part its refractoriness to treatment.

**Conclusions:**

The understanding of both central and peripheral mechanisms that contribute to the development of pain is of major importance in order to propose more effective treatments for brachial plexus avulsion-related pain. This review focuses on the current understanding about the occurrence of neuropathic pain in these patients and the role played by peripheral and central mechanisms that provides insights into its treatment.

**Summary:**

Pain after brachial plexus avulsion involves both peripheral and central components; thereby it is characterized as a mixed (central and peripheral) neuropathic pain syndrome.

## Review

### Historical aspects

The first cases of traumatic nerve root avulsion were described in the eighteenth century. In 1872, Duchenne de Boulogne described paralysis of the muscles innervated by the rostral roots of the brachial plexus (BP) in a patient with obstetric paralysis [1]. In 1874 Erb [2] described the anatomical landmark located 2–3 cm superior to the clavicle at the convergence of the ventral primary rami of the C5 and C6 spinal nerves (Erb’s point) and showed that rupture of these structures was a common presentation of brachial plexus injury (BPI). Klumpke [3] described the complete injury of all elements of the BP and suggested that the presence of the Claude-Bernard-Horner sign indicated injury to the first thoracic root or its sympathetic branches. In 1947, Murphey et al. [4] described the radiological findings that were observed in patients with brachial plexus avulsion with myelography and set up the first diagnostic workups based on imaging studies.

### Classification

**Figure 1:**
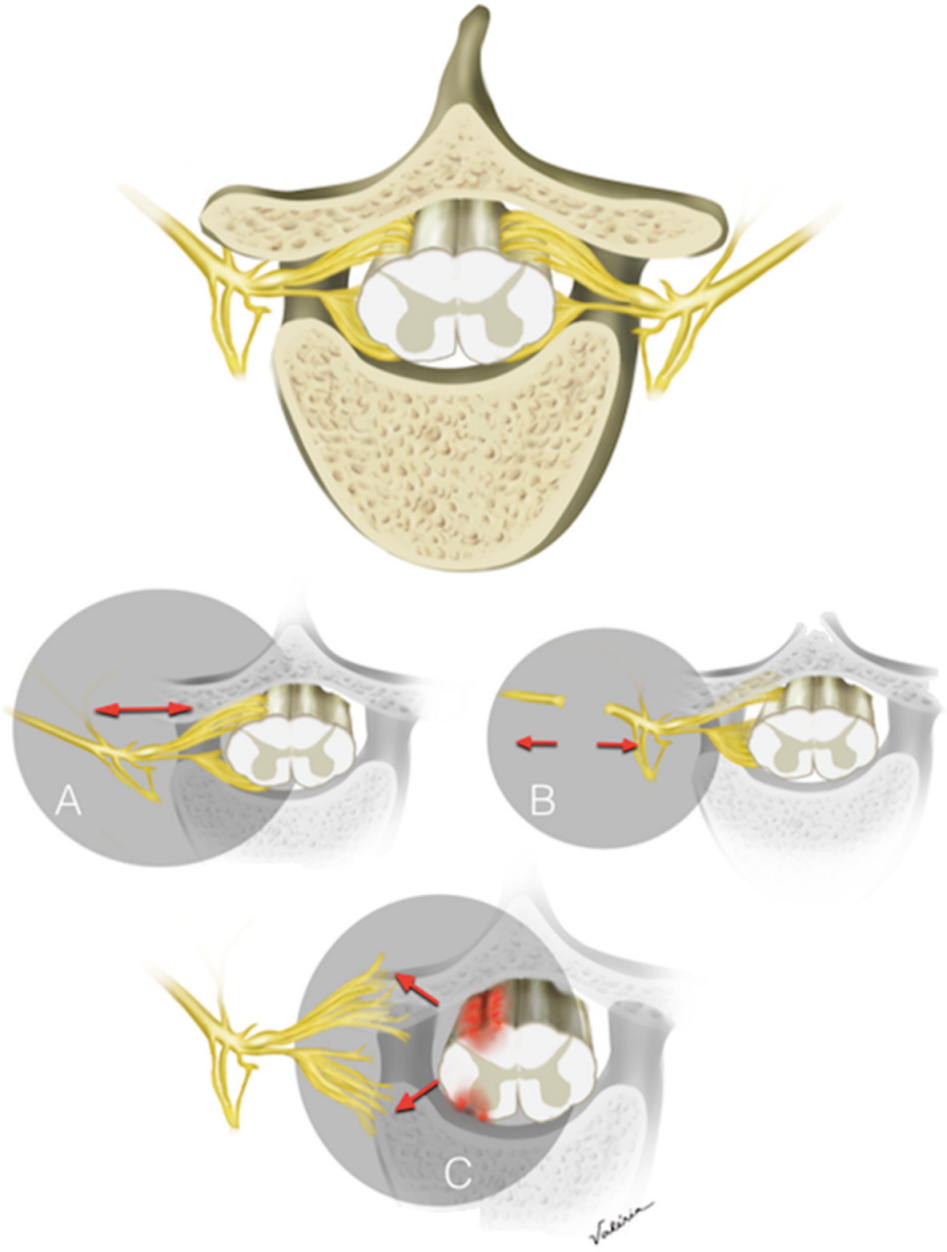
Types of Brachial plexus injuries. Representative illustration of normal vertebral body, spinal cord and dorsal root ganglia. **A**: Stretch lesion with no macroscopic lesion to fibers, **B**: post-ganglionic lesions, **C**: pre-ganglionic lesion (avulsion).

Brachial plexus avulsion (BPA) was classified by Parry [5] as one of three main types of traction injuries that affect the brachial plexus that are defined as the pre-ganglionic disruption of the nerve roots from the spinal cord (Figure 1). Traction is, in fact, the most common mechanism that produces avulsion, although compression or crushing can also occur. BPA can also be considered as a specific type of brachial plexus injury (BPI) and can be classified as open or closed regarding the presence of an open wound as reason for the lesion. BPAs are always located superior to the clavicle [6]. BPAs are preganglionic, meaning that the injury involves the severing of axons that form the spinal nerve roots and include the proximal axons of the primary afferent and efferent rootlets. This takes place between the dorsal root ganglia and the spinal cord [7].

In pre-ganglionic lesions, the sensory and motor nerves are disconnected from the spinal cord, but the cell bodies of sensory fibers (dorsal root ganglia) are preserved. Such an injury maintains peripheral nerve integrity, therefore electrical studies can be performed to determine the nerve structures, location and severity of the lesion in all its complexity [6,8-10]. Pain severity seems to be related to the number of roots avulsed [11]. The differentiation between pre- and post-ganglionic BPIs is of paramount importance and has prognostic and surgical implications. The gold standard for distinguishing between pre- and post-ganglionic lesions is the direct exploration of the rootlets during surgery. Imaging studies such as CT myelography and MRI have lower accuracy for making this distinction (85 and 52%, respectively [12]). BPA usually requires nerve transfer (usually the intercostal nerve) to the distal stump of the intact nerve, whereas postganglionic injuries are commonly grafted [6,7]. Nerve transfer is the connection of a functioning nerve (of lesser functional importance) to a distal stump of a functionally relevant avulsed nerve structure in patients with disability due to BPA, whereas nerve grafting, on the other side, involves the use of a graft (normally a nerve auto-graft) to bridge proximal to the distal stumps [13].

### Pain after Brachial Plexus Avulsions

Pain is the symptom that has the greatest negative impact on the quality of life of patients with plexus avulsion [12] and is usually caused by associated trauma to the musculoskeletal system (e.g. tendon ruptures) and traumatic neuropathy, among other causes [14]. Similar to other conditions where the primary lesion is of neurological origin, BPA patients are subject to additional pain syndromes such as nociceptive pain and complex regional pain syndrome, among others. Nociceptive pain is associated with tissue injury that results in activation of pain receptors (i.e., nociceptors), often related to inflammation, which can lead to a peripheral hyperexcitability of the nociceptive system. Although poorly documented, there are many sources of nociceptive pain potentially associated with brachial plexus injury, including myofascial pain (Araújo et al., in submission), tendinitis, postoperative lesions, muscle overload due to unbalanced muscle activity and joint pain. Neuropathic pain (NeP) is associated with abnormal neuronal functioning due to a lesion or disease affecting the somatosensory system (SS) [15,16]. Lesions of the SS are usually associated with negative phenomena, such as loss of touch sensitivity (mechanical hypoesthesia), decrease in pain after pinprick (hypoalgesia) or loss of cold or warm sensitivity (thermal hypoesthesia). However, lesions of the SS also cause positive symptoms, associated with spontaneous neuronal firing or amplification of the signal [15,16].

In BPIs, pain is reported from 67% [15] to 71% [17] of patients. The prevalence of neuropathic pain in these patients is high, reaching up to 95% in some series [18,19]. According to Ciaramitaro [19], among patients with BPIs, those who had avulsions (BPAs) reported neuropathic pain more frequently than the patients whose injury involved other portions of the brachial plexus. Very few studies have assessed the prevalence and the characteristics of pain syndromes other than NeP in patients following BPA [20,21].

Despite the peripheral contributions to the NeP present after BPA, there is a large body of evidence that also supports the role of central injury-related mechanisms in the genesis of pain in these patients. Central sensitization (CS), which is a classical mechanism leading to the amplification and chronification of pain [22-25], increases the gain of the injured somatosensory system and leads to increased perception to painful stimulus (hyperalgesia), decrease in thresholds (allodynia) and increase in the central processing of peripheral inputs leading to secondary hyperalgesia, which typically extends beyond the denervated area [24]. Different types of brachial plexus lesions lead to CS, such as ligation, crushing and avulsion. However, different lines of evidence suggest that BPA leads to central changes that are not frequently seen in other peripheral nerve lesions [26,27] and are narrowly related to lesions of the central nervous system associated with avulsion.

In many instances, avulsions lead to neurophysiological and clinical modifications that were closer to spinal cord lesions than to post-ganglionic injuries. For example, experimental models of BPA elicited significant and longer-lasting bilateral mechanical and cold allodynia when compared to crushing and ligation models [28]. Importantly, signs of neuropathy (and not only CS) could be detected at distant sites from lesion, both in ipsi and contralateral paws, without signs of autotomy, in contrast to what is seen in models of chronic constriction injury, partial sciatic tight ligation or selective lumbar spinal neurectomy. These changes may be related to a direct lesion of segmental spinal cord tissue that occurs after the abrupt disconnection of spinal roots during BPA [16,24,28-30] (Figure 1). Neuronal loss occurs at various levels, both on the same side and contralateral to the lesion [31]. Neuronal death secondary to inflammation and trauma itself may contribute to this phenomenon via the release of cytokines and chemokines release, glial cells activation and neuronal apoptosis [32,33]. Decreases in interhemispheric inhibition could explain similar symptoms reported by amputees that may share some similarities with BPA [34-37] as will be discussed below.

In this review we will focus on the available clinical, neurophysiological and molecular evidence suggesting that BPA patients with neuropathic pain have a mixed (central and peripheral) type of NeP syndrome. BPA uniquely involves the area of transition between the central and peripheral nervous system and this could account for some clinical peculiarities related to the marked refractoriness to conventional treatments and greater negative impact on quality of life [5] seen in these patients.

### Literature search strategy

A search of literature published from 1996 to 2014 on the prevalence, clinical characterization, pain mechanisms and treatment options after BPA was conducted by using open databases (Google Scholar and Pubmed). Key words included the following: plexus avulsion, neuropathic pain, chronic pain, trauma, brachial plexus, treatment. Study selection: the review included studies with relevant information to the understanding of the mechanisms, clinical presentation and treatment options for BPA. Data extraction: data retained included the pathophysiology, prevalence, clinical profile, type of pain associated with BPA and treatment options related to BPA. Data synthesis: a narrative synthesis was employed to express the results.

## Conclusions

### Peripheral and central mechanisms of pain in the region of avulsion

It is known that lesions of the *substantia gelatinosa* and Lissauer Tract (LT) are associated with the occurrence of pain in cases of BPA [38]. The posterior horn of the spinal cord (PHSC) and LT are the first integration centers of the primary sensory afferents in the neuroaxis [34]. The LT is located at the apex of PHSC and its fibers are distributed longitudinally along the spinal cord [35]. About one third of its fibers are primary afferents projecting, rostral or caudally for one or more spinal segments [36]. The other fibers originate in the PHSC itself [37,39,40]. Both the medial and lateral sides of the LT contain propriospinal fibers, but only the medial component is associated with nociceptive transmission [41].

It seems that both the medial and lateral components of the LT play an important role in modulating a normal overlapping of receptive fields from different dorsal roots. As the lateral LT plays an inhibitory effect, its lesion leads to a net facilitation of the local neurons causing expansion of receptive fields mainly after the third day of lesion [42]. The reversal of the symptoms after injections of strychnine into these damaged areas is evidence that this mechanism is likely to be post-synaptic [43]. Also, it has been demonstrated that avulsions lead to lesions of the medial aspect of the LT. In monkeys avulsion led to atrophy of the medial aspect of the *substantia gelatinosa* at the level of the lesion and contraction of the respective dermatome [43]. In cats, avulsion also lead to a more pronounced injury to the medial aspect of the LT and the lateral dorsal column, with subsequent gliosis of the *substantia gelatinosa*, leading to sensory and receptive field changes after injury that were closer to an actual spinal cord lesion than to rhizotomy [44]. Apart from these structures, avulsions were related to dorsolateral fasciculus lesions and long-term reduction in myelinated fibers in the PHSC [30]. There is evidence that hyperactive PHSC neurons, under the influence of disinhibited lateral LT, are largely responsible for the pain in cases of root avulsion where there has been a loss of the primary afferent fibers [45-50]. A further support to this idea is that lesions to the LT and PHSC after Lissauer’s tractotomy (Dorsal Root Entry Zone procedure: “DREZ-tomy”) lead to significant pain relief in instances of BPA. In animals, the autotomy behavior (discomfort behavior) is also abolished after lesion of both LT and PHSC [43-48]. Animals treated exclusively with sensory ganglionectomy have more autotomy behavior than those undergoing ganglionectomy and LT + PHSC, or even LT lesion alone [49].

Aside from hyperactivity, some of the pathological changes identified after avulsion include spontaneous neuronal activity and enlargement of the receptive fields of a specific subgroup of PHSC neuronal population – for instance, those located in laminae IV to VI. It is known that the avulsion of myelinated fibers causes damage to the pericornual layer and *substantia gelatinosa* fibers, where presynaptic inhibition of primary afferents [51] occurs. In cases of plexus avulsions, impairment of the PHSC interneurons and the pain gate mechanisms proposed by Melzack and Wall [52] could occur, resulting in expansion of spontaneous neuronal activity along the spinal cord [53] and facilitation of the activity of neurons that give rise to the reticulospinal tract [54]. Neurons that have lost their primary afferents located in laminae IV to VI of PHSC begin to react, at least partially, to stimuli conveyed by the surviving intact afferent nerve fibers and develop new receptive fields that take the place of the pre-existing ones [55]. A prolonged expansion of the receptive fields of neurons in the PHSC occurs, which is attributed to the anatomical involvement of rostrocaudal tract fibers representing suppressor or inhibitory supraspinal neurons (dorsal ventromedial medulla descending fibers) and its consequent loss of inhibitory control [44]. Cats undergoing plexus avulsion showed marked reduction in SP in superficial (I, II) and deeper (V) lamina, while somatostatin was decreased in lamina II. These changes were followed by a decrease in enkephalin concentration in lamina I, II and V [53]. It has been hypothesized that enkephalinergic neurons would have inhibitory effects upon lamina I and II neurons (pre-synaptic inhibition) and lamina V neurons projecting to the thalamus (post-synaptic inhibition). The depletion of somatostatin interneurons in lamina II and V would also contribute to this loss in inhibition. Concomitantly, denervation hypersensitivity due to loss of SP neurons may ensue both in the superficial (I, II) and deeper (V) laminae. It has been hypothesized that DREZ-tomy would destroy from lamina I thorough V and would terminate this abnormal hyperactivity [53].

Compared to ligation and crushing neuropathic pain models, BPA has also been shown to cause a longer lasting mechanical hyperalgesia and cold allodynia, which were present bilaterally, and not confined to the body area supplied by the injured cervical roots [29]. This is evidence to support a central, spinal cord injury-related phenomena caused by avulsion, which is responsible for more positive pain-related signs and sensory changes in body areas that could not be explained by a simple peripheral mechanism or CS. In fact, a microrecording study showed that the neuronal discharge behavior of the posterior horn in patients with recurrent pain due to BPA were closer to those seen in patients with spinal cord lesions and spasticity [42]. Additionally, it has been shown that there was more neuronal hyperactivity in patients with BPA than in those with other peripheral nerve injuries or spasticity [56]. Cats start to display autotomy manifested by the self-mutilation of the skin in the dermational fields of the distal extremity that is associated with the area of deafferentation, within hours of root avulsion. Occasionally, they also scratched the intact contralateral limb, which means that the plexus avulsion generated abnormal sensations bilaterally [44].

Additionally, the trigger zones observed in patients with root avulsion appear to be due to the prolonged increase in excitatory activity that originated in areas with normal innervation and that were distant from the deafferentation fields [44]. It is likely that the involvement of the LT and a more significant degeneration observed in the PHSC deep laminae justify the differences observed between root avulsion or rhizotomy in animal models. In another study performed in cats, there was hyperactivity and expansion of the receptive fields in neurons located in lamina V of the PHSC in animals, which underwent rhizotomy or root avulsion. In cases of rhizotomy, hyperactivity in the lamina V and in the superficial laminae of the PHSC remained for several months, while, in cases of root avulsion, the neurons of the superficial laminae remained relatively quiet and, in the lamina V, regular activity of high frequency began three weeks after the procedure [57]. Neuronal hyperactivity was observed more in the PHSC on the affected side (by rhizotomy or root avulsion) than on the contralateral side [58]. This means that, in cases of root avulsion, the stimuli located in non-adjacent ipsilateral areas on the affected limb greatly facilitate the neurons located on the surface of the spinal cord segments, which suffered from deafferentation. These segments undergo an increase in the receptive fields, synaptic reorganization and biochemical and cellular alterations that may or may not remain stable [29,59].

The permanent neuronal hyperactivity observed in the PHSC in peripheral neuropathy cases may be due to the preservation and persistence of the connection between the sensory ganglia and the CNS neurons, a condition that allows neuronal activation by ganglionic ectopic potentials [60]. It suggests that in BPA there is a lack of inhibition because of the impairment of rostrocaudal neuronal inhibition in the CNS, caused by avulsion but not by other peripheral neuropathies [57]. Molecular studies (immunohistochemistry and *in situ* hybridization) further support this idea. It has been shown that early genes such as c-Jun and growth-related proteins such as GAP-43 are up-regulated when axotomy takes place distal to the dorsal root ganglia. On the other hand, when a lesion occurs proximally, such as in the case of BPA, the reverse occurs, and no up regulation of these genes is triggered. This further supports the importance of central damage with a poor regenerative response compared to distal injuries in the development of autotomy [61,62]. These data lend support for the presence of the phenotypical patterns seen after central lesions of afferent sensory neurons and not just the functional changes expected to occur centrally due to peripheral deafferentation. As explained, these phenotypical patterns involve less collateral sprouting and poorer regenerative response compared to those observed in lesions distal to the DRG [61].

The above data suggest that avulsion leads to molecular, anatomical, biochemical, sensory, and neurophysiological changes that are different from simple rhizotomy, and include central lesions to the spinal cord, at least up to the medial aspect of the LT. As we have been discussing, secondary central plastic changes occur after sensory deafferentation to the CNS [63-65] and phenomena such as central sensitization is widely known to take place and could account for receptive field changes and sensory threshold modifications. However, nerve root avulsions still present particularities that include the anatomical disconnection to the sensory ganglia and lesion of spinal cord structures that probably account for its unique clinical presentation. BPA is associated with a much higher incidence of neuropathic pain when compared to other peripheral neuropathies such as diabetic polyneuropathy (11-26%) [66] and CNS conditions such as stroke (8%) [67] and multiple sclerosis (55%) [68]. Additionally, it presents highly refractory pain [19,65,69].

### Cortical mechanisms of pain

Some patients who suffer from plexus avulsion perceive painful symptoms and movement sensations in the affected limb [5]. This phenomenon is called Phantom Limb Pain (PLP) and occurs in 54–85% of amputees [70-72]. Phantom limbs are perceived not only after amputation [73], but also after nerve avulsion [71] (39,3% after BPA) [5], spinal cord injury and in about 20% of children with congenital limb aplasia [74]. The self representation of the phantom limb can resemble the healthy member or mimic images of the limb itself with its previous disease [70,75]. These phenomena are interpreted as the re-organization of the cortical structures related to the regions that suffered the avulsion or amputation. These cortical areas seem to undergo an invasion of adjacent representation areas such as that responsible for tongue sensitivity [69]. Interestingly, PLP is frequent after limb amputation and BPA, but is rare after lesions anatomically restricted to peripheral nerves, such as polyneuropathy or nerve root injuries not associated with avulsion.

Anatomical and clinical findings have been put forward to link PLP mechanisms to lesions in the peripheral nerve system, such as the neuroma formation and the presence of sweating and vasoconstriction at the onset or during the painful symptoms (autonomic nerve system manifestations). Moreover, the presence of mechanical, chemical and electric irritation in the stump and the improvement observed after anesthetic nerve blockades proximal to the stump with long-term pain relief also contribute to this idea [76,77]. On other hand, there is evidence against the peripheral theory, suggesting that central mechanisms play a more important role in BPA-related PLP: [1] the lack of pain improvement after rhizotomy and/or anesthetic blockade of the autonomic system; [2] the lack of dermatome distribution of the pain and [3] the rarity of PLP in children under six years old [76-79]. Also other evidence reinforce the CNS contribution to PLP symptoms, such as the long-lasting aspect of this disease, the dispersion of the pain from the original phantom area to other previously healthy areas in the body and the inhibition of pain after the therapeutic stimulation of CNS structures [80,81].

Neuroplasticity involves all the nervous system, specially the cerebral cortex, which is extremely important in cases of BPA [82]. There is constant neuroplasticity and rearrangement of cortical representation maps in cortical and subcortical areas. Areas that suffer from deafferentation usually undergo cortical and subcortical changes, which occur both immediately after the injury or progressively develop in the time [63,64]. These areas commonly suffer reduction in cortical representation while the adjacent areas that maintain their afferent input stimuli enlarge their respective receptive fields. For instance, Merzenich [83,84] observed that, after median nerve transection of owl and squirrel monkeys, cortical representation of the dorsum of the radial hand and of digits 1, 2 and 3, coupled with representation of the ulnar bordering glabrous skin surface expanded onto cortical areas that previously represented skin surface innerved by the median nerve, which were silenced after nerve transection. Melzack [80] postulated that abnormal activity of neuronal brain circuits was related to phantom limb sensations. Studies using transcranial magnetic stimulation reported that muscles located near the amputation stump presented higher amplitude motor evoked potentials than homologous muscles in the contralateral intact side. Additionally, there was an increased blood flow in temporal, parietal and frontal regions of the brains of the patients suffering a severe PLP crisis [85,86]. These findings lend some support to the conclusion that there is a relationship between amount of cortical reorganization and the PLP magnitude [71]. There is evidence that PLP is related to a genetic predisposition combined with previous environmental exposure to painful sensations. One good example of this is the extremely low frequency of PLP in children with limb agenesis [74,87] and the fact that half of the children that had suffered amputation before they were six years-old had phantom sensations. Other important evidence suggests that past painful experiences in the affected limb predisposed the individual to develop PLP after an amputation. Pre-amputation pain has been related to an increased risk of PLP. This is especially true in pediatric population [88] and vascular amputees [89]. However, the relationship between pre-amputation pain and PLP is not linear and may not remain present when patients are followed by longer periods of time [90].

Falconer [91] described that phantom limb pain does not significantly improve after operations in the peripheral nervous system (rhizotomy), while it does after central procedures (cordotomy and DREZ). Unlike rhizotomy, during the DREZ operation, neurosurgeons use the posterolateral sulcus as landmark to access the root entry zones and perform radiofrequency lesions longitudinally several segments above and bellow the avulsed area [92,93]. On the other hand, for the cordotomy procedure, the electrode is positioned anterior to the dentate ligament where lies the spinothalamic tract [94]. Interestingly, the DREZtomy has provided significant long-term improvement of phantom limb and BPA related pains [95]. Moreover, in patients who suffered from brachial plexus avulsions (BPA) related to traumatic amputation, there was a sustained 70% improvement in the pain intensity in 66.7% of the patients who underwent DREZ operation. Similar results were found after the same procedure in BPA patients who underwent limb amputation to relieve refractory pain, suggesting that positive results after Lissauer’s tratotomy were independent of the time when amputation occurred [96]. The effects of DREZ operation rely on the elimination of hyperactive neurons in the PHSC [97]. Some authors argue that, regarding BPA pain phenotype clusters, paroxysmal pain is more associated to hyperactive neurons in the PHSC and continuous pain relates particularly to supraspinal structures [42,98,99]. This is possibly the reason why DREZtomy has been shown to be more effective against paroxysmal pain than against continuous pain after BPA [97,99]. In contrast, electric stimulation of the motor cortex (with epidural electrodes on the precentral gyrus) has shown better results for continuous BPA pain possibly because it modulates supraspinal structures and its descending inhibitory effects on the remaining PHSC cells after the avulsion [97]. Therefore, it seems that BPA patients benefit more from procedures that target structures in the PHSC and other CNS structures than peripheral procedures, especially when there is evidence of associated PLP [95,96]. This is further support for the idea that BPA patients have a more complex and refractory pain syndrome when compared to strictly peripheral neuropathies, and central mechanisms other than central sensitization are likely to play a role in its genesis and maintenance.

Regarding the pharmacological therapies, there is a lack of evidence-based treatment for NeP in BPA, as is the case for posttraumatic neuropathies. A recent meta-analysis reported no clear benefit in the use of antidepressant, anticonvulsants and any other drug class (NMDA inhibitor, cannabinoids). Only opioids showed some positive (weak) effect, with number needed to treat ranging from 2.7 to 36 [100].

### Summary

Pain is a common symptom after BPI, affecting 71% to 78% of patients. In most of these cases (67%) the pain is predominantly neuropathic. However, when patients with only BPA are analysed, pain is ominous and highly refractory to the usual treatments. It is probable that BPA affects mainly the CNS structures that can suffer influences from the PNS, giving rise to a mixed neuropathic pain syndrome with major central components.

BPA leads to specific pathological changes that are different from changes observed in rhizotomy and other strictly “peripheral” neuropathies. Avulsions compromise part of the spinal cord (LT, PHSC, and possibly part of the dorsolateral fasciculus) leading to an initial decrease of the activity of superficial neurons and the *substantia gelatinosa*, followed by a late onset of heightened high frequency activity in deeper layers of the PHSC (lamina V). Abnormalities also include ectopic neuronal activity and central sensitization. Clinically patients may present with severe burning of paroxysmal pain often located in areas outside the involved nerve roots, and commonly associated with PLP, which may occur after lesions to the CNS. These changes suggest that in BPA patients lesions affect both central and peripheral neural components, leading to a mixed neuropathic pain syndrome, which could account for some of its characteristics.

BPA pain is largely refractory to the usual pharmacological treatments and is frequently managed by neuromodulation and neuroablative techniques with variable success. A broader understanding of its mechanisms, specifically taking into account the peculiarity of the pain syndromes concerning the associated central lesions, will pave the way for more accurate management of BPA patients.

## Abbreviations

BPI: Brachial plexus injury
BPA: Brachial plexus avulsion
CT: Computed tomography
MRI: Magnetic resonance imaging
NeP: Neuropathic pain
SS: Somatosensory system
CNS: Central nervous system
LT: Lissauer tract
PHSC: Posterior horn of the spinal cord
PLP: Phantom limb pain
DREZ: Dorsal root entrance zone
